# The Characteristics of Cognitive Impairment in ALS Patients Depend on the Lateralization of Motor Damage

**DOI:** 10.3390/brainsci10090650

**Published:** 2020-09-19

**Authors:** Umberto Manera, Laura Peotta, Barbara Iazzolino, Antonio Canosa, Rosario Vasta, Francesca Palumbo, Maria Claudia Torrieri, Luca Solero, Margherita Daviddi, Maurizio Grassano, Cristina Moglia, Marco Pagani, Adriano Chiò, Marco Cavallo

**Affiliations:** 1 ALS Centre, Department of Neuroscience “Rita Levi Montalcini”, University of Torino, 10126 Turin, Italy; umberto.manera@gmail.com (U.M.); lapeo92@gmail.com (L.P.); barbara.iazzolino@gmail.com (B.I.); antoniocanosa85@gmail.com (A.C.); sarovasta@gmail.com (R.V.); fra-palumbo@libero.it (F.P.); mariaclaudia.torrieri@gmail.com (M.C.T.); lucasolero91@gmail.com (L.S.); margherita.daviddi@gmail.com (M.D.); grassano.maurizio@gmail.com (M.G.); cristina.moglia@unito.it (C.M.); adriano.chio@unito.it (A.C.); 2 SC Neurologia 1U, AOU Città della Salute e della Scienza of Torino, 10126 Turin, Italy; 3 Institute of Cognitive Sciences and Technology, CNR, 00185 Rome, Italy; marco.pagani@istc.cnr.it; 4 Department of Medical Radiation Physics and Nuclear Medicine, Karolinska University Hospital, 17164 Stockholm, Sweden; 5 Faculty of Psychology, eCampus University, 22060 Novedrate, Italy

**Keywords:** amyotrophic lateral sclerosis, neuropsychological evaluation, frontotemporal dementia, hemispheric lateralization, language, visuospatial abilities

## Abstract

(1) Background: Cognitive features of patients with amyotrophic lateral sclerosis (ALS) have never been specifically analyzed according to the lateralization of motor impairment. In the present study we investigated the cognitive performances of ALS patients to describe the relationship between motor and cognitive dysfunction, according to site and side of disease onset. (2) Methods: Six-hundred and nine ALS patients underwent a comprehensive neuropsychological evaluation at diagnosis in Turin ALS Centre Tests included—mini-mental state examination (MMSE), frontal assessment battery (FAB), trail-making test A/B (TMT A-B), digit span forward and backward (digit span FW/digit span BW), letter fluency test (FAS), category fluency test (CAT), Rey auditory verbal learning test (RAVLT), Babcock story recall test (BSRT), Rey-Osterrieth complex figure test (ROCFT), Wisconsin card sorting test (WCST), Raven’s coloured progressive matrices (CPM47). Cognitive performances of patients, grouped by side and site of onset, were statistically compared using *z*-scores, as appropriate. (3) Results: Bulbar patients and bilateral spinal onset patients (S_bil_) were generally characterized by lower cognitive performances in most neuropsychological tests, when compared to patients with lateralized onset (right-side spinal onset, S_ri_ and left-side spinal onset, S_le_). Digit span backward and visual memory task (ROCFT) median *z*-scores were significantly higher, reflecting a better cognitive performance, in S_ri_ patients when compared to bulbar/S_bil_ patients, while verbal memory tasks (RAVLT and BRST) resulted in significantly higher scores in S_le_ patients. Our results are in keeping with hemispheric functional lateralization of language and visuospatial abilities. (4) Conclusions: In ALS patients, as in other neurodegenerative diseases, we found a direct relationship between lateralized motor and cognitive features.

## 1. Introduction

Amyotrophic lateral sclerosis (ALS) is a neurodegenerative disorder that primarily involves all voluntary motor functions, through progressive degeneration of upper and lower motor neurons [1]. It is characterized usually by a focal onset in limbs or bulbar muscles, where upper and lower motor signs are maximal [2], spreading through contiguous or network pathways [3]. In limb-onset patients, the site of onset is usually lateralized, especially in patients with upper limb onset, and has been related to limb dominance with a high concordance for side of onset and handedness [4]. Cognitive impairment is a frequent additional feature of ALS [5,6], being mainly related to frontotemporal cortical neuron degeneration, such as motor impairment. There are indications that cognitive function may worsen during disease progression [7], with a correlation to motor dysfunction and clinical stages [8].

Despite this general correlation between motor and cognitive impairment, the patterns followed by neurodegeneration to spread across are not fully understood but seem to maintain a certain degree of lateralization [3]. An area that has been generally neglected in ALS is the notion that some cognitive functions, which can be impaired in the disease, are functionally lateralized, in particular language and visuospatial abilities [9].

The aim of the present study was to analyze the performance of a cohort of ALS patients in different cognitive domains according to the site and side of disease onset, and to identify the presence of specific cognitive patterns related to the lateralization of motor impairment.

## 2. Materials and Methods

### 2.1. Data Collection

The study population included all ALS cases diagnosed between 2010 and 2018 who underwent neuropsychological evaluation at the Turin ALS Center. Site of onset, side of onset, date of onset, sex, hand dominance, and years of formal education were collected using data recorded in the Piemonte and Valle D’Aosta register for ALS (PARALS, [10]). All neuropsychological evaluations were performed at diagnosis (diagnosis-evaluation interval < 3 months). The battery included a series of tests aimed at evaluating executive function, memory, visuospatial function, and language, selected according to the diagnostic criteria for the behavioral variant of frontotemporal dementia [11] and the amyotrophic lateral sclerosis frontotemporal dementia (ALS-FTD) consensus criteria [12]. Specifically for this study we considered mini-mental state examination (MMSE), Wisconsin card sorting test (WCST), trail-making test A (TMT-A) and B (TMT-B) and (TMT-B-A), digit span forward (digit span FW) and backward (digit span BW), letter (FAS) and category (CAT) fluency test, Rey auditory verbal learning test (RAVLT), Babcock story recall test (BSRT), Rey-Osterrieth complex figure test (ROCFT), Raven’s colored progressive matrices (CPM47), and frontal assessment battery (FAB) [13]. The study design was approved by the Ethical Committee of the Azienda Ospedaliero-Universitaria Città della Salute (prot. n. 0036344). Patients signed a written informed consent. Data will be available upon request by interested researchers.

### 2.2. Cognitive Categorization

Patients’ cognitive status was classified, according to the revised ALS-FTD Consensus Criteria, [12] into five categories: (1) patients with ALS with a frontotemporal dementia (FTD) syndrome (ALS-FTD); (2) patients with ALS with behavioral impairment (ALSbi); (3) patients with ALS with cognitive impairment (ALSci); and (4) patients with ALS with combined cognitive and behavioral impairment (ALScbi), which includes patients who fulfilled criteria for both ALSci and ALSbi. Patients who did not fit into these categories were classified as cognitively normal (ALS-CN).

### 2.3. Side of Onset Classification

Patients’ history was carefully evaluated to classify patients by side of onset, considering neurological symptoms such as dysphagia, dysarthria, muscle atrophy, strength and dexterity reduction to attribute disease onset. Muscle cramps and fasciculation were not considered. Patients were classified into four categories: bulbar onset patients, right-side spinal onset patients (S_ri_), left-side spinal onset patients (S_le_) and bilateral spinal onset patients (S_bil_). S_bil_ included mainly patients with predominant upper motor neuron predominant phenotype [14], whose symptoms were referred to start in both upper and lower limbs simultaneously.

### 2.4. Statistical Analysis

In order to make proper comparisons between different tests, we derived adjusted raw scores for age, sex and education using the Italian normative scores. Then we calculated *z*-scores, using as reference the study population. Differences in discrete and continuous variables were analyzed using the *χ*^2^ test, Kruskall–Wallis test and Mann–Whitney U test, with the implementation of Bonferroni’s correction for multiple comparisons, respectively. A *p* value < 0.05 was considered significant.

Data were analyzed using IBM SPSS Statistics for Windows, version 26.0. (Armonk, NY, USA: IBM Corp.) and Graph Pad Prism 8.4.2.

## 3. Results

We collected neuropsychological evaluations from 609 ALS patients. In the whole population, 97.8% of patients were right-handed and we did not stratify the analysis according to handedness. The main descriptive statistics are summarized in Table 1, classified according to the site/side of onset (bulbar onset, S_ri_, S_le_ and S_bil_). We also further detailed in Table S1 the exact number of patients considered for each neuropsychological test.

The study population was not significantly different from PARALS population [10] (see Table S2). Compared to patients with lateralized (S_ri_ and S_le_) spinal onset, patients with bulbar onset were older (Mann–Whitney-U test *p* = 0.017) and predominantly female (bulbar 58.7% vs. spinal 34.5%, chi-square test *p* < 0.001), as expected, while also patients with bilateral spinal onset were more frequently older (bilateral vs. lateralized spinal onset, Mann–Whitney-U test *p* = 0.020). Considering proportions, right-side was the most frequent side of onset in the upper limb onset, while left-side and bilateral onset were more frequent in patients with lower limb onset.

**Figure 1:**
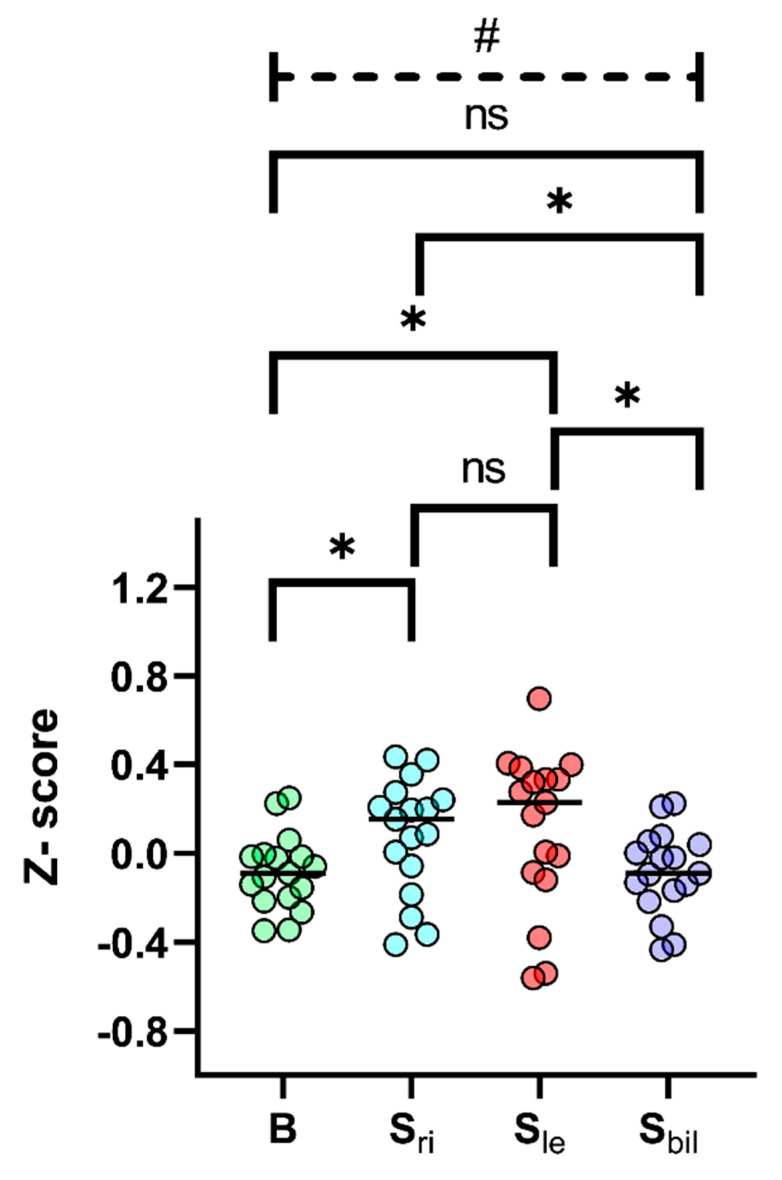
Median *z*-scores for all neuropsychological tests subdivided by site of onset (bulbar vs. spinal onset) and side of onset for spinal onset patients (right-side vs. left-side vs. bilateral onset). # Kruskal–Wallis test *p* < 0.05; * Mann–Whitney U test (with Bonferroni’s correction) *p* < 0.05. The black horizontal lines identified the median *z*-score for all tests. B, bulbar onset patients; S_ri_, right-side spinal onset; S_le_, left-side spinal onset; S_bil_, bilateral spinal onset.

According to ALS-FTD consensus criteria [12], more than a half of bulbar onset patients were classified as cognitively impaired, with 18.8% of patients diagnosed with ALS-FTD. Considering adjusted scores for single neuropsychological tests, we found significant differences according to the site and side of onset in most of them. Bulbar onset patients were generally characterized by lower scores, usually followed by S_bil_ (i.e., in MMSE, FAS, CAT, FAB, RAVLT delayed recall, BSRT and CPM47). Using *z*-scores, we compared all the different tests in the whole study population. All median *z*-scores values were furtherly compared using appropriate non-parametric tests: bulbar onset patients resulted having a significantly worse cognitive impairment than spinal onset patients (Mann–Whitney U test comparison among median *z*-scores, *p* = 0.031, see also Figure S1). Interestingly, after stratification for side of onset, patients with lateralized onset (S_le_ more than S_ri_) obtained better scores in almost all tests, obtaining significantly different results from both bulbar and S_bil_, which showed the same *z*-score median value (see Figure 1).

To evaluate the role of specific neuropsychological tests in determining such differences, we compared *z*-scores for each test in the overall population (see Table 2).

A subset of tests (MMSE, letter and category fluency) obtained significantly different results among patients with bulbar onset and patients with lateralized spinal onset (both S_ri_ and S_le_), but not among bulbar onset patients and S_bil_. Interestingly, bulbar and S_bil_ did not show any significant differences in median *z*-scores after multiple comparisons.

Digit span (both forward and backward), ROCFT copy and CPM47 obtained significantly better results in S_ri_ patients compared to bulbar onset patients. Moreover, S_ri_ patients had significantly better scores compared to S_bil_ in letter fluency, while S_le_ did not differentiate significantly.

S_le_ were instead characterized by high *z*-scores in neuropsychological tests that evaluated verbal function, particularly verbal memory, such as RAVLT delayed recall (S_le_ vs. bulbar, *p* = 0.030 and S_le_ vs. S_ri_, *p* = 0.022) and BSRT immediate recall (S_le_ vs. bulbar, *p* = 0.004) and delayed recall (S_le_ vs. bulbar, *p* < 0.001, S_le_ vs. S_ri_, *p* = 0.033, and S_le_ vs. S_bil_, *p* = 0.006). Compared to bulbar onset patients, S_le_ showed better scores also in TMT B (*p* = 0.029) and TMT B-A (*p* = 0.023).

## 4. Discussion

Our findings point out that cognitive features of ALS patients are related to motor impairment and follow hemispheric lateralization, suggesting a possible disease spreading or simultaneous degeneration of highly interconnected frontal and precentral neurons. In keeping with previous studies, we have found that patients with bulbar onset are generally more cognitively impaired than those with spinal onset [8]. However, when we subdivided patients not only according to the site of onset (bulbar vs. spinal), but also according to the side of onset, we found that spinal patients with symmetric motor impairment have significantly worse cognitive performance than those with lateralized damage, and appear to have a cognitive dysfunction similar to bulbar patients. ALS is characterized by a focal onset, followed by a regional spreading that generally maintains a certain grade of asymmetry [15]. We used motor dysfunction as a proxy for contralateral hemispheric damage in order to evaluate the ability of neuropsychological tests in assessing cognitive functions that are highly lateralized, such as verbal memory and visuospatial abilities. Since the description of the ALS-FTD spectrum [16], the interconnection between motor and cognitive impairment has become one of the main topics in the ALS research field. Recent findings have pointed out that the extension of motor impairment, measured using King’s and MiToS clinical staging systems, is correlated with cognitive decline [8]. Despite this, the complex mechanisms underlining their relationship are far from being fully understood and probably only in vivo neurodegeneration monitoring through neuroimaging could help to solve this problem [17].

The role of lateralization of motor damage in ALS has been widely studied [2,4], but, to the best of our knowledge, at present, no study has compared the initial asymmetrical features of motor impairment to specific cognitive tasks in a large cohort of ALS patients [18]. In Parkinson’s disease (PD), which is another neurodegenerative disorder characterized by both motor and cognitive impairment, the asymmetry of extrapyramidal signs is included in the diagnostic criteria [19], but its underlying mechanisms have not been fully understood yet [20]. The dominant motor cortex, which is typically related to handedness, is characterized by a greater connectivity compared to the non-dominant side [21]. Moreover, transcranial magnetic stimulation studies [22] in healthy people have demonstrated a greater neuronal excitability in the dominant hemisphere, confirming a great difference in neurophysiology of the cerebral hemispheres. In ALS, handedness more than footedness has been related to the side of symptom onset and motor impairment [4], influencing, mainly for upper motor neuron features, the subsequent spread of the disease [23]. Additionally, in PD the dominant right hand is the most common site of onset [20] and when affected, it is correlated with global worse cognitive dysfunction [24]. On the contrary, left handedness and the non-dominant side of onset are associated with milder phenotypes [25]. In our ALS cohort, we only partly confirmed these observations: the subgroup S_le_, with their dominant left hemisphere less affected, showed the highest median *z*-score in the majority of neuropsychological tests, especially in the most frequently used, such as MMSE, TMT and verbal function tests.

Considering the absence of significant lateralization of FAS and CAT, we can speculate that those tasks are not specifically influenced by left hemisphere impairment, confirming the results of a previous study in which ALS patients with verbal fluency deficits were characterized by bilateral dorsolateral prefrontal cortex, lateral and medial premotor cortex, insular cortex, and thalamus dysfunction [26].

On the contrary, we found a strong lateralization in neuropsychological tests more related to verbal memory, namely RAVLT and BSRT, with S_le_ showing significantly higher *z*-scores when compared to all other groups. S_le_’s left hemisphere relative preservation could justify a better performance of these subjects on verbal memory tasks. This result confirms previous diffusion-tensor imaging findings that correlate left hemisphere white matter tract integrity (specifically left perforant pathway and left uncinate fasciculus) with better performances at RAVLT and BSRT [27]. Additionally, the TMT B and B-A *z*-scores resulted to correlate with a left hemisphere sparing, with better performances obtained by S_le_ when compared to bulbar patients. Our findings confirmed a recent review [28], which found evidence of an association between left-lateralized lesion sites (rostral anterior cingulate, left dorsomedial prefrontal cortex, left insular cortex, non-specific lesion sites within left prefrontal, insular, temporal and parietal cortex) and poor performance on the TMT-B.

S_ri_ *z*-scores for digit span backward and forward were significantly higher than bulbar patients, while S_le_ and S_bil_ did not differ significantly, these findings are in keeping with previous reports that correlate with both bilateral and right frontal areas (particularly right dorsolateral prefrontal cortex: DLPFC, inferior parietal lobule: IPL and anterior cingulate cortex: ACC) with short-term verbal retention tasks [29,30]. In S_ri_, the relative sparing of right cerebral frontal areas, such as DLPFC, could explain higher performances for digit span backward and forward.

Considering ROCFT copy and CPM47, we observed a significant difference between bulbar onset and S_ri_ patients. These results show that left-side and bilateral impairment are crucial in determining a worse performance in these tasks, in part confirming what is observed in PD, where a left-side predominant involvement is related to specific cognitive decline in visuospatial performances [31,32]. To our knowledge, no previous data on ROCFT and CPM47 lateralization in the ALS population were published.

This study has some limitations. First, the definition of the side of onset was based on patient clinical history; more objective data, such as the measure of strength with specific rating scale (i.e., Medical Research Council: MRC), would have improved this information. Second, our results are based on clinical and neuropsychological data, a correlation with neuroimaging data (MRI, PET) would be useful to identify the anatomical and functional correlates of our findings. Third, by considering neuropsychological evaluation at diagnosis and patients’ history, we could not clarify the causality or the order of involvement of cognitive and motor dysfunction, but we only assessed their compresence.

## 5. Conclusions

We described, for the first time in ALS patients, a direct relationship between lateralized motor and cognitive features, using a properly sized cohort. Our findings are in keeping with evidence in other neurodegenerative diseases, such as PD. They could be useful in many research fields, such as neuropathology and neuroimaging, but also in clinical practice, helping to stratify patients into different cognitively susceptible categories to monitor disease progression more effectively and organize care management properly.

## Figures and Tables

**Table 1:** Descriptive statistic of amyotrophic lateral sclerosis (ALS) patients subdivided by site of onset (bulbar vs. spinal onset) and side of onset for spinal onset patients (right-side vs. left-side vs. bilateral onset).

Site/Side of Onset		Bulbar Onset	Right-Side Spinal Onset	Left-Side Spinal Onset	Bilateral Spinal Onset	Total	
		***n* (%*^r^*)**	***n* (%*^r^*)**	***n* (%*^r^*)**	***n* (%*^r^*)**	***n* (%*^r^*)**	
Total		218 (35.8)	174 (28.6)	105 (17.2)	112 (18.4)	609 (100.0)	
		***n (*%*^c^)***	***n (*%*^c^)***	***n (*%*^c^)***	***n (*%*^c^)***	***n (*%*^c^)***	***p ^#^***
Sex							**<0.001 *^#^***
Male		90 (41.3)	115 (66.1)	63 (60.0)	78 (69.6)	346 (56.8)	
Female		128 (58.7)	59 (33.9)	42 (40.0)	34 (30.4)	263 (43.2)	
Site of Onset							**<0.001 *^#^***
Bulbar Onset		218 (100)	-	-	-	218 (35.8)	
Upper Limbs Onset		-	107 (61.5)	45 (42.9)	22 (19.6)	174 (28.6)	
Lower Limbs Onset		-	67 (38.5)	60 (57.1)	90 (80.4)	217 (35.6)	
Cognitive Classification							**<0.001 *^#^***
ALS-CN		102 (46.8)	108 (62.1)	68 (64.8)	61 (54.5)	339 (55.7)	
ALSci		15 (6.9)	23 (13.2)	11 (10.5)	11 (9.8)	60 (9.8)	
ALSbi		43 (19.7)	25 (14.4)	14 (13.3)	25 (22.3)	107 (17.6)	
ALScbi		17 (7.8)	9 (5.2)	6 (5.7)	8 (7.1)	40 (6.6)	
ALS-FTD		41 (18.8)	9 (5.2)	6 (5.7)	7 (6.3)	63 (10.3)	
		**Median**	**Median**	**Median**	**Median**	**Median**	***p***
		**(IQR)**	**(IQR)**	**(IQR)**	**(IQR)**	**(IQR)**	
Age at Onset (Years)		70.0	67.0	66.0	70.5	69.0	0.007 *
		(62.7–76.0)	(58.0–74.0)	(59.0–73.0)	(61.2–75.7)	(60.0–74.0)	
Education (Years)		8.0	8.0	8.0	8.0	8.0	0.221 *
		(5.0–11.0)	(5.0–11.0)	(5.0–11.0)	(5.0–11.0)	(5.0–12.0)	
Neuropsychological Test	*n*	**Median**	**Median**	**Median**	**Median**	**Median**	***p***
		**(IQR)**	**(IQR)**	**(IQR)**	**(IQR)**	**(IQR)**	
MMSE	603	27.4	28.0	28.5	27.4	27.8	<0.001 *
		(25.4-–29.2)	(26.5–30.0)	(27.3–30.0)	(26.0–29.4)	(26.3–30.0)	
Letter Fluency Test	584	25.4	31.3	31.0	27.4	28.6	<0.001 *
		(19.2-–33.1)	(23.5–38.6)	(23.6–39.1)	(20.9–34.8)	(21.9–35.4)	
Category fluency test	455	17.2	19.8	19.3	18.8	19.3	0.002 *
		(14.0–21.0)	(16.5–23.1)	(16.5–22.5)	(15.5–23.9)	(15.5–22.3)	
FAB	505	14.7	15.2	15.3	14.7	14.9	0.048 *
		(11.9–16.3)	(13.9–16.7)	(13.5–17.1)	(12.4–16.5)	(13.3–16.5)	
Digit Span FW	538	5.5	5.8	5.5	5.7	5.5	0.026 *
		(4.7–6.1)	(5.1–6.5)	(4.9–6.2)	(5.1–6.2)	(4.9–6.3)	
Digit Span BW	442	3.7	4.1	3.8	3.7	3.9	0.013 *
		(3.3–4.3)	(3.5–4.7)	(3.5–4.3)	(3.2–4.3)	(3.4–4.4)	
TMT-A	540	43.0	39.5	35.0	37.5	39.0	0.176 *
		(28.0–76.0)	(23.3–63.5)	(26.0–52.0)	(25.0–54.0)	(26.0–62.0)	
TMT-B	540	94.0	82.0	61.0	76.5	79.0	0.024 *
		(43.0–275.0)	(35.5–144.5)	(38.0–139.0)	(43.3–180.0)	(40.0–179.8)	
TMT-B-A	540	59.5	41.0	28.0	39.0	45.0	0.013 *
		(13.3–155.0)	(10.5–90.0)	(7.0–94.5)	(16.0–120.5)	(12.0–121.0)	
RAVLT-ir	281	38.0	38.3	41.9	38.0	38.7	0.083 *
		(30.1–44.1)	(31.6–45.5)	(34.3–46.6)	(34.0–43.2	(32.8–44.5)	
RAVLT-dr	281	7.8	7.2	8.9	7.8	7.8	0.004 *
		(4.8–10.3)	(5.1–10.4)	(7.2–11.2)	(5.7–9.9)	(5.6–10.2)	
BSRT-ir	289	5.5	5.8	6.4	5.3	5.7	0.005 *
		(4.2–6.6)	(4.7–6.9)	(5.2–7.8)	(4.1–6.8)	(4.5–6.8)	
BSRT-dr	279	6.0	6.5	7.3	6.1	6.4	<0.001 *
		(4.5–7.2)	(4.8–7.8)	(6.1–8.0)	(4.7–7.4)	(4.9–7.6)	
ROCFT-copy	426	30.8	32.4	32.0	30.8	31.5	0.005 *
		(23.1–33.8)	(29.3–35.1)	(29.5–34.4)	(26.3–34.1)	(26.9–34.5)	
ROCFT-dr	422	11.0	12.0	12.0	10.6	11.5	0.140 *
		(7.5–15.9)	(8.8–16.6)	(7.4–15.4)	(7.0–14.3)	(7.7–15.8)	
CPM47	574	27.4	29.5	29.3	27.5	28.5	0.012 *
		(23.0–31.5)	(25.2–32.1)	(26.1–31.9)	(21.7–31.5)	(24.3–31.8)	
WCST	187	80.0	85.3	79.4	78.8	82.8	0.724 *
		(62.8–92.8)	(58.5–98.7)	(35.1–96.5)	(43.5–94.1)	(52.8–93.9)	

%*^r^* Raw subgroup percentage; %*^c^* column subgroup percentage; ^#^ Chi-square test; * Kruskal–Wallis test. *p*-Value < 0.05 were considered as significant and written in bold. (ALS-FTD, ALS with a frontotemporal dementia; ALSbi, ALS with behavioral impairment; ALSci, ALS with cognitive impairment; ALScbi, ALS-CN, ALS with normal cognition; MMSE, mini-mental state examination; WCST, Wisconsin card sorting test; TMT-A, TMT-B, TMT B-A, trail-making Test A and B and B-A; digit span FW and BW, digit span forward and backward (digit span BW); RAVLT, Rey auditory verbal learning test; BSRT, Babcock story recall test; ROCFT, Rey-Osterrieth complex figure test; CPM47, Raven’s colored progressive matrices; FAB, frontal assessment battery; ir, immediate recall; dr, delayed recall; IQR, interquartile range).

**Table 2:** *Z*-score comparison according to site and side of onset (bulbar onset, B vs. right-side spinal onset, S_ri_ vs. left-side spinal onset, S_le_ vs. bilateral spinal onset, S_bil_) for different neuropsychological tests.

Neuropsychological Test	Bulbar (B)	Spinal Right-Onset (S_ri_)	Spinal Left-Onset (S_le_)	Spinal Bil-Onset (S_bil_)	B vs. S_ri_ vs. S_le_ vs. S_bil_	B vs. S_ri_	B vs. S_le_	B vs. S_bil_	S_ri_ vs. S_le_	S_ri_ vs. S_bil_	S_le_ vs. S_bil_
	Median (IQR)	Median (IQR)	Median (IQR)	Median (IQR)	*p ^#^*	p *	p *	p *	p *	p *	p *
MMSE	−0.02	0.19	0.40	−0.02	**<0.001**	**0.009**	**<0.001**	0.999	0.999	0.179	**0.003**
	(−0.78–0.66)	(−0.35–0.96)	(−0.07–0.96)	(−0.55–0.74)							
Letter fluency test	−0.35	0.20	0.17	−0.17	**<0.001**	**<0.001**	**0.001**	0.999	0.999	**0.036**	0.053
	(−0.93–0.36)	(−0.53–0.88)	(−0.51–0.93)	(−0.77–0.53)							
Category fluency test	−0.34	0.07	−0.01	−0.10	**0.002**	**0.002**	**0.046**	0.160	0.999	0.999	0.999
	(−0.88–0.36)	(−0.47–0.63)	(−0.47–0.53)	(−0.64–0.77)							
FAB	0.06	0.24	0.28	0.08	**0.048**	0.092	0.479	0.999	0.999	0.327	0.916
	(−0.95–0.64)	(−0.23–0.78)	(−0.36–0.91)	(−0.76–0.69)							
Digit Span Forward	−0.11	0.15	−0.09	0.05	**0.026**	**0.035**	0.999	0.187	0.999	0.999	0.999
	(−0.95–0.53)	(−0.54–0.87)	(−0.66–0.65)	(−0.54–0.59)							
Digit Span Backward	−0.14	0.21	−0.12	−0.14	**0.013**	**0.009**	0.999	0.999	0.405	0.149	0.999
	(−0.66–0.38)	(−0.40–0.82)	(−0.41–0.43)	(−0.78–0.45)							
TMT A	0.22	0.29	0.38	0.33	0.176	0.579	0.310	0.982	0.999	0.999	0.999
	(−0.47–0.53)	(−0.21–0.62)	(0.03–0.57)	(−0.01–0.59)							
TMT B	0.26	0.36	0.54	0.41	**0.024**	0.215	**0.029**	0.999	0.999	0.999	0.734
	(−1.24–0.69)	(−0.16–0.75)	(−0.11–0.73)	(−0.45–0.69)							
TMT B-A	0.20	0.41	0.56	0.43	**0.013**	0.083	**0.023**	0.999	0.999	0.999	0.531
	(−0.91–0.73)	(−0.15–0.76)	(−0.21–0.80)	(−0.51–0.70)							
RAVLT-ir	−0.09	−0.06	0.33	−0.09	0.083	0.999	0.112	0.999	0.480	0.999	0.178
	(−0.94–0.57)	(−0.78–0.72)	(−0.50–0.84)	(−0.52–0.47)							
RAVLT-dr	0.00	−0.19	0.33	−0.02	**0.011**	0.999	**0.030**	0.999	**0.022**	0.999	0.053
	(−0.92–0.74)	(−0.83–0.77)	(−0.19–1.03)	(−0.65–0.63)							
BSRT-ir	−0.06	0.09	0.41	−0.13	**0.005**	0.297	**0.004**	0.999	0.390	0.999	0.095
	(−0.67–0.50)	(−0.41–0.65)	(−0.18–1.09)	(-0.71–0.59)							
BSRT-dr	−0.01	0.27	0.70	0.04	**<0.001**	0.790	**<0.001**	0.999	**0.033**	0.999	**0.006**
	(−0.80–0.63)	(−0.65–0.96)	(0.08–1.07)	(−0.69–0.76)							
ROCFT-copy	0.22	0.43	0.39	0.22	**0.005**	**0.007**	0.227	0.999	0.999	0.129	0.678
	(−0.76–0.62)	(0.04–0.80)	(0.06–0.70)	(−0.35–0.66)							
ROCFT-dr	−0.15	0.01	0.01	−0.22	0.140	0.337	0.999	0.999	0.999	0.228	0.999
	(−0.72–0.64)	(−0.52–0.74)	(−0.74–0.55)	(−0.80–0.38)							
CPM47	−0.02	0.35	0.32	0.00	**0.012**	**0.050**	0.062	0.999	0.999	0.340	0.335
	(−0.79–0.70)	(−0.40–0.81)	(−0.24–0.78)	(−1.03–0.71)							
WCST TOT	0.25	0.42	0.23	0.21	0.724	0.999	0.999	0.999	0.999	0.999	0.999
	(−0.31–0.66)	(−0.45–0.85)	(−1.21–0.78)	(−0.94–0.71)							

^#^ Kruskal–Wallis test. * Mann–Whitney U test with Bonferroni’s correction for multiple comparisons. *p*-Value < 0.05 were considered as significant and written in bold (MMSE, mini-mental state examination; WCST, Wisconsin card sorting test; TMT-A, TMT-B, TMT B-A, trail-making test A and B and B-A; digit span FW and BW, digit span forward and backward (digit span BW); RAVLT, Rey auditory verbal learning test; BSRT, Babcock story recall test; ROCFT, Rey-Osterrieth complex figure test; CPM47, Raven’s colored progressive matrices; FAB, frontal assessment battery; ir, immediate recall; dr, delayed recall).

## Supplementary Materials

The following are available online at https://www.mdpi.com/2076-3425/10/9/650/s1, Table S1: Number of patients who underwent different neuropsychological tests grouped by site/side of onset, Table S2: Comparison between PARALS population (2010-2018 period) and Turin ALS Centre who underwent neuropsychological evaluation. Figure S1: Median *z*-scores for all neuropsychological tests subdivided by site of onset (bulbar vs. spinal onset) * Mann-Whitney U test (with Bonferroni’s correction) *p* < 0.05.
