# Supplementary material for: The Characteristics of Cognitive Impairment in ALS Patients Depend on the Lateralization of Motor Damage
Source: Brain Sci. 2020 Sep 19;10(9):650. doi: 10.3390/brainsci10090650 (PMC7563934; doi:10.3390/brainsci10090650)
Supplement: Supplementary file 1 [file brainsci-10-00650-s001.pdf]

Table S1. Number of patients who underwent different neuropsychological tests grouped by site/side of onset.

Site/side of onset	Bulbar onset	Spinal right-side onset	Spinal left-side onset	Spinal bilateral onset	Total	
	n (%r)	*n (%r)*	*n (%r)*	*n (%r)*	*n (%c)*	
Total	218 (35.8)	174 (28.6)	105 (17.2)	112 (18.4)	609 (100.0)	
Neuropsychological Test						*p**
MMSE	216 (35.8)	171 (28.4)	104 (17.2)	112 (18.6)	603 (99.0)	0.999
Letter Fluency Test	201 (34.4)	171 (29.3)	104 (17.8)	108 (18.5)	584 (95.9)	0.965
Category fluency test	151 (33.2)	137 (30.1)	83 (18.2)	84 (18.5)	455 (74.7)	0.833
FAB	179 (35.4)	142 (28.1)	88 (17.4)	96 (19.0)	505 (82.9)	0.993
Digit Span FW	188 (34.9)	153 (28.4)	97 (18.0)	100 (18.6)	538 (88.3)	0.983
Digit Span BW	148 (33.5)	127 (28.7)	82 (18.6)	85 (19.2)	442 (72.6)	0.864
TMT-A	191 (35.4)	156 (28.9)	95 (17.6)	98 (18.1)	540 (88.7)	0.997
TMT-B	191 (35.4)	156 (28.9)	95 (17.6)	98 (18.1)	540 (88.7)	0.997
TMT-B-A	191 (35.4)	156 (28.9)	95 (17.6)	98 (18.1)	540 (88.7)	0.997
RAVLT-ir	89 (31.7)	73 (26.0)	59 (21.0)	60 (21.4)	281 (46.1)	0.289
RAVLT-dr	89 (31.7)	73 (26.0)	59 (21.0)	60 (21.4)	281 (46.1)	0.289
BSRT-ir	90 (31.1)	80 (27.7)	59 (20.4)	60 (20.8)	289 (47.5)	0.400
BSRT-dr	84 (30.1)	77 (27.6)	60 (21.5)	58 (20.8)	279 (45.8)	0.228
ROCFT-copy	150 (35.2)	120 (28.2)	79 (18.5)	77 (18.1)	426 (70.0)	0.962
ROCFT-dr	150 (35.5)	118 (28.0)	77 (18.2)	77 (18.2)	422 (69.3)	0.980
CPM47	205 (35.7)	164 (28.6)	100 (17.4)	105 (18.3)	574 (94.3)	0.999
WCST	64 (34.2)	58 (31.0)	31 (16.6)	34 (18.2)	187 (30.7)	0.933

%r Raw percentage; %c Column percentage of patients who underwent the specific test compared to the whole study population; p* Chi-square test between single test population vs. whole study population.

Table S2. Comparison between PARALS population (2010-2018 period) and Turin ALS Centre who underwent neuropsychological evaluation.

	**PARALS**	**Study group**	***p***
	n = 1332	*n* = 609	
Age at onset	69.9 (61.7–76.1)	69.0 (60.0–74.0)	0.082 *
Education	7.5 (5.0–11.5)	8.0 (5.0-12.0)	0.360 *
Sex			0.805 #
Male	748 (56.2)	346 (56.8)	
Female	584 (43.8)	263 (43.2)	
Site of Onset			
Bulbar Onset	427 (32.1)	218 (35.8)	0.273 ##
Upper Limbs Onset	363 (27.3)	174 (28.6)	
Lower Limbs Onset	511 (38.4)	217 (35.6)	
Other/undetermined	31 (2.3)	0 (0.0)	

* Mann-Whiney U test; # Fisher's exact test; ## Chi-square test.

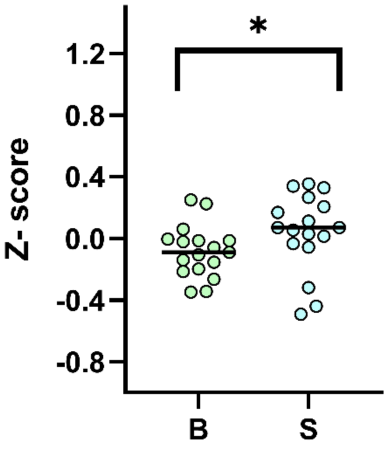

Figure S1. Median *z*-scores for all neuropsychological tests subdivided by site of onset (bulbar vs. spinal onset) * Mann-Whitney U test (with Bonferroni's correction) $p < 0.05$.
